# Intussusception

**Published:** 1907-06-22

**Authors:** 


### Diseases of Children.

### INTUSSUSCEPTION.

Early diagnosis of the occurrence of intussusception is imperative if treatment is to prove efficacious. The following case exhibits most of the typical features of the affection:—

A healthy, well-nourished, breast-fed boy, six months old, vomited in the evening and seemed to have abdominal pain. Castor oil was given. During the night blood was passed, together with a little fæcal matter. The child was pale and languid, and remained so until he was seen next day about 1 P.M. On examination per rectum an elongated swelling was felt above and to the right of the umbilicus. About half an ounce of dark blood was passed just after. The abdomen was lax, and the swelling could be felt indistinctly between the fingers in front and the thumb in the right loin. There was no local tenderness, and apparently little pain. Laparotomy was done 19 hours after the onset. The intussusception was a double one—primarily enteric, secondarily ileo-cæcal. The last part was tightly constricted, was reduced with difficulty, and was very dark in colour, but not gangrenous. After the operation the child was breast-fed every three hours. The bowels acted freely next day, the stools being loose, green, and free from blood. For a few days after this the stools were green, and contained a little mucus and curds. The child took the breast well. There was some irregular fever, up to 103° F., for eight days. For the first few days after operation the child looked pale, and the eyes were sunken. He then gradually improved. The stitches were removed 15 days after the operation, and the child was discharged three days later in excellent health.

The above case affords several valuable lessons. It was a male. For some unknown reason males are more often affected than females; in the proportion of 2 to 1 (Holt), 77 to 30 (Hirschsprung), 16 to 6 (Sherren). He was under a year old. Two-thirds of the cases occur in the first year of life, and the affection is most common at the age of three to nine months. He was breast-fed. Hirschsprung states that 85 per cent. of 46 cases under one year old were entirely breast-fed. Indigestible food is sometimes the exciting cause, and in the present instance the mother subsequently confessed she had given the child sponge-cake the day before the onset. Noticeably, too, the affection is most common about Christmas and the New Year. The intussusception was of the usual variety—namely, ileo-cæcal or ileo-colic. It was not, however, a simple ileo-cæcal one, being primarily enteric, starting a few inches above the cæcum and subsequently invaginated into the cæcum.

In the matter of diagnosis the symptoms were not absolutely characteristic. There was no history of antecedent intestinal trouble; no severe and paroxysmal abdominal pain; and not much vomiting. On the other hand, there was a definitely sudden onset with some pain and vomiting, marked collapse, and the passage of blood per anum. Pain is not always present, nor is it necessarily severe. Perhaps it is only felt at the onset. Usually it is colicky, paroxysmal, and severe for a day or two. Vomiting occurs in 80 to 90 per cent. It is most marked at the onset, and may not be repeated until the late stages, or it may continue throughout. Blood and mucus in the stools are characteristic. Blood may be passed at the onset, but more often not until after 6 to 12 hours. A little fæcal matter may be passed at first. After that the constipation is complete, and no flatus is passed. A tumour is present in 80 to 90 per cent., though it will often be missed if the child is not examined under an anæsthetic. Both a rectal and a bimanual examination must be made. In the early stages the tumour will not be felt in quite 50 per cent. of all babies under a year. Often it is very ill-defined. After a time it reaches the rectum, and may even protrude as a purplish lump, liable to be mistaken for a prolapse of the rectum, piles, or polypus. At first the abdomen is relaxed. Possibly there is a little tenderness and rigidity over the tumour. Later it becomes tympanitic. Much importance must be attached to the "abdominal facies" which develops in a few hours. There is marked prostration, sudden in development and out of proportion to the symptoms. The babe looks ill, collapsed, pale, cold, with sunken eyes and anxious aspect. The pulse is small, feeble, and unduly frequent. The temperature is often subnormal. In the present case the diagnosis was based on the sudden onset, the vomiting, the prostration, the passage of blood, and the presence of an ill-defined tumour.

The proper treatment of these cases is immediate operation. Spontaneous reduction is possible, and has been reported. Reduction on bimanual manipulation, with the finger in the rectum, has been recorded. Inflation, irrigation, and injection should only be resorted to if for any reason an operation is out of the question. Such measures will reduce colic intussusceptions, and possibly ileo-cæcal ones in the early stages. Unfortunately it is impossible to be sure that reduction is complete. If it is not, all the symptoms return in a few hours, and the prospects of successful operation are much decreased, because of the prolonged injury to the gut.

The prognosis of laparotomy is excellent, provided it is done soon—that is, within 36 hours from the onset. Fagge records 15 recoveries out of 19 babies under one year. Clubbe reported 63 recoveries out of 100 cases in children. Of Hess's collected cases 69 out of 74 recovered under operation, when one or no previous attempt had been made to cure by irrigation; 83 out of 119 recovered after operation, preceded by attempts at cure by irrigation; and only 34 out of 60 recovered under non-operative treatment.

After operation, breast feeding should be continued when possible. A few minim-doses of tincture of opium may be needed. Fever, as in the above case, is sometimes due to absorption of toxins from the damaged gut.

